# Early Detection System of Vascular Disease and Its Application Prospect

**DOI:** 10.1155/2016/1723485

**Published:** 2016-11-30

**Authors:** Huan Liu, Hongyu Wang

**Affiliations:** Department of Vascular Medicine, Peking University Shougang Hospital, Beijing 100144, China

## Abstract

Markers of imaging, structure, and function reflecting vascular damage, integrating a long time accumulation effect of traditional and unrecognized cardiovascular risk factors, can be regarded as surrogate endpoints of target organ damage before the occurrence of clinical events. Prevention of cardiovascular disease requires risk stratification and treatment of traditional risk factors, such as smoking, hypertension, hyperlipidemia, and diabetes. However, traditional risk stratification is not sufficient to provide accurate assessment of future cardiovascular events. Therefore, vascular injury related parameters obtained by ultrasound or other noninvasive devices, as a surrogate parameter of subclinical cardiovascular disease, can improve cardiovascular risk assessment and optimize the preventive treatment strategy. Thus, we will summarize the research progress and clinical application of early assessment technology of vascular diseases in the present review.

## 1. Introduction

In the year of 2010, a concept of “ideal cardiovascular health” was put forward, including ideal healthy behavior, such as status of smoking, body mass index, physical activity and diet, and ideal healthy factors, such as blood pressure, blood glucose, and blood lipids [1]. However, the truth was that, in the 2012 global ten main death causes, vascular diseases such as ischemia heart disease and stroke were the first two according to the report of World Health Organization. In addition, the recent Chinese cardiovascular report indicated that there were 290 million cardiovascular disease patients, 270 million hypertension patients, and 7 million stroke patients, and 1 person in every 5 may be a cardiovascular disease patient [2]. In face of such a huge disease burden, prevention and treatment of vascular related diseases are equally important. And from what have been known, the main pathophysiological mechanism of lesions in heart, brain, kidney, and other organs is the blood vessels that supply the above organs suffering from arteriosclerosis, atherosclerosis, stenosis, and occlusion, which lead to impaired vascular function and structure and further lead to the occurrence of adverse cardiovascular events, such as coronary heart disease, stroke, peripheral arterial occlusion, and even sudden death [3]. Therefore, during the progression of vascular disease from risk factors (such as hypertension, abnormal glucose metabolism, and dyslipidemia) to vascular damage to the target organ damage, before the occurrence and development of the end-stage events, it will be of great importance to recognize surrogate endpoints which can accurately and effectively reflect the clinical endpoints. Furthermore, the surrogate endpoints can be delayed and even reversed by lifestyle and drug intervention. Therefore, early evaluation of surrogate endpoints and intervention to prevent or even reverse the occurrence of adverse vascular events have become a hot research topic in the world.

## 2. Vascular Endothelial Function

Vascular endothelial cells as a layer of single cell interface between blood flow and vessel wall, responding to the stimulation of body fluid, the nerve, especially the hemodynamics, can regulate vascular tone by the synthesis and release of vasoactive substances, regulate platelet function, inflammatory response, and vascular smooth muscle cell growth and migration, and play an important role in the pathogenesis of vascular diseases [4, 5]. Vascular endothelial dysfunction is a systemic disorder characterized by reduced nitric oxide or nitric oxide bioavailability and is the first step in the natural process of arteriosclerosis and atherosclerosis, directly relating to numerous vascular diseases. However, endothelial dysfunction can be reversible, which is different from atherosclerosis. Therefore, it has important significance for the prevention and system management and effective intervention by early clinical detection of vascular disease. Vascular endothelial function can be of diagnostic, prognostic, and predictive value [6]; it can be assessed by invasive procedures such as coronary angiography and noninvasive measurement techniques such as flow-mediated dilation (FMD) and finger end volume recording.

### 2.1. Coronary Angiography

Acetylcholine stimulation by coronary angiography is the traditional method of vascular endothelial function detection [7]. It is the gold standard for the detection of vascular endothelial function by precise measurement of coronary blood flow using Doppler guide wire. But the method is an invasive examination and the operation is difficult and time consuming with high cost. At present, it is difficult to carry out widely in clinical practice.

### 2.2. Flow-Mediated Dilation (FMD)

Doppler ultrasound equipment was used to measure the baseline and cuff released diameter of brachial artery, and FMD was calculated as the rate of brachial artery diameter changes. It reflects the flow-mediated endothelial nitric oxide (NO) release function and is considered as a commonly used noninvasive method. The method is simple, noninvasive, economical and accurate, reproducible, and clinically beneficial for the promotion of early vascular lesion detection [8]. FMD as a surrogate endpoint to assess cardiovascular risk is widely used in clinical studies. Research suggested that brachial artery FMD was a predictor of cardiovascular events [9] and was a predictor of future target organ damage in low-risk patients with essential hypertension [10]. But FMD detection is highly dependent on operator expertise and cannot rule out nerve interference and environmental factors. In addition, ultrasonic imaging is not suitable for high-precision detection, and thus it limits the wide clinical applications of FMD.

### 2.3. Pulse Amplitude Tonometry- (PAT-) Reactive Hyperemia Index (RHI)

FMD reflects NO-mediated function of conduit brachial artery and is measured by Doppler ultrasound apparatus. Measurement of digital pulse amplitude tonometry (PAT) is based on a technique similar to that of FMD. It uses photoplethysmography to measure pulse wave amplitude (PWA) in the fingers reflecting small vessel microcirculation function and gets the reactive hyperemia index (RHI). RHI is defined as the ratio of postdeflation to baseline pulse amplitude in the hyperemic finger divided by that in the contra-lateral finger [11]. Studies indicated that RHI independently correlated with future cardiovascular events and added incremental clinical significance for risk stratification [12, 13]. RHI can also predict patients with ischemic heart disease and it improved risk stratification when added to traditional risk factors [14, 15]. However, the finger-tip devices are for one use only, so there is moderate disposable cost for studies [16].

Both brachial FMD and RHI are independent predictors of cardiovascular events and all-cause mortality and both with similar prognostic magnitude [17, 18]. RHI reflects the vasodilator function in the microvasculature of the fingers whereas FMD reflects the vasodilator function in large conduit vessels. FMD serves as an index of NO-mediated endothelium-dependent vasodilator function. Unlike FMD measured at the brachial artery, RHI is not entirely caused by NO and only 50% of it was blocked when a nitric oxide synthase specific blocker (L-NAME) was infused into the brachial artery before PAT measurement [19]. The divergence between brachial and digital vascular function emphasizes the possibility that vasodilation may vary significantly with vessel size and location. Arterial physiology across vascular beds may have implications for the utilization of the PAT and FMD test to measure vascular function [20]. Studies showed that FMD had differing relations with cardiovascular risk factors and were nearly uncorrelated with RHI. These results suggest that FMD and RHI provide distinct information regarding vascular function in conduit versus smaller digital vessels [20, 21].

Therefore, whether the prognostic values of these 2 methods are independent of each other and whether an endothelial function-guided strategy can provide benefit in improving cardiovascular outcomes need further research to determine [18].

## 3. Evaluation of Arterial Stiffness

Arterial stiffness is mainly determined by larger arteries, because the thoracic aorta and abdominal aorta contribute the greatest to arterial buffering function. Elastic properties of conduit arteries vary along the arterial tree, with proximal arteries being more elastic and distal ones stiffer. In addition, aging and blood pressure are the major determinants of arterial stiffness, which causes a decrease in elastic protein synthesis and an increase in degradation, while the synthesis of type 3 and type 1 collagen is increased and the degradation is reduced [22]. Studies have shown that changes in arterial structure can be quantified in accordance with vascular stiffness and epidemiological studies have indicated that arteriosclerosis is a risk factor for cardiovascular disease progression and is a strong independent predictor of cardiovascular disease and total mortality [23]. Many techniques can be used as assessment of vessel, including assessment of arterial stiffness, as well as the evaluation of subclinical atherosclerosis. This review will carry on a summary on the application of arterial sclerosis detection technologies according to the method of detection, clinical significance, and application value.

### 3.1. Pulse Wave Velocity (PWV)

PWV is a common indicator of arterial stiffness and can be obtained by measurement of distance and pulse wave transit time between two points of vessels [8]. It can be measured by ultrasonic equipment or blood vessel detection equipment automatically and can measure different arterial segments, such as carotid femoral artery, carotid radial artery, carotid dorsal artery, and brachial ankle artery. Application of noninvasive arteriosclerosis detection device can automatically detect PWV, with the advantages of simple operation and good repeatability, but is vulnerable to the impact of immediate blood pressure. Among them, carotid femoral pulse wave velocity (CF-PWV) is considered to be the gold standard for assessment of large artery stiffness [23]. Studies have shown that higher arterial stiffness was significantly correlated with increased subclinical disease burden of coronary artery, lower extremity artery, and cerebral artery [24]. It is not only a manifestation of vascular aging but also a predictor of cardiovascular disease risk [25–27] and is an independent predictor of functional outcome in patients with acute ischemic stroke [28]. In addition, Framingham study found that in people with higher aortic stiffness the risk of cardiovascular disease increased by 48%, and adding PWV to the standard risk model can improve the risk prediction value of cardiovascular events (such as myocardial infarction, unstable angina, heart failure, and stroke) [25].

### 3.2. Cardio Ankle Vascular Index (CAVI)

CAVI is a new evaluation index of arterial stiffness in recent years and can also be measured by means of arteriosclerosis detection device. Compared with PWV, CAVI does not depend on the immediate effect of blood pressure. CAVI comes from the stiffness coefficient *β* and can be calculated by the electrocardiogram, phonocardiogram, and brachial ankle artery pulse waveform and pulse waveform records and is mainly related to aortic stiffness and compliance [29]. A series of studies have found that CAVI as a new marker of vascular health was closely related to arterial injury in patients with hypertension, diabetes, metabolic syndrome, and blood lipid disorder and became a useful tool for health screening because of the blood pressure dependence [30–35]. In addition, higher CAVI was also associated with lower executive function in older adults [36], and CAVI was also an effective predictor of cardiovascular events in obese patients [37]. In addition, a new classification of vascular health has regarded CAVI as an important evaluation index of vessel in clinical practice [38].

### 3.3. Other Assessment of Arterial Stiffness

Central aortic pressure (CAP) can be measured by invasive cardiac catheterization and noninvasive applanation tonometry of the radial or carotid artery [39, 40]. CAP waveforms were determined by a mathematical transformation of the radial waveform or directly ascertained from the carotid arterial pressure waveforms obtained by noninvasive applanation tonometry [41, 42]. Most used was noninvasive radial tonometry to acquire waveforms to derive CAP. And CAP can be estimated noninvasively using an automated tonometer device and showed good validity as evaluated by the catheter method [43]. CAP can reflect the load of left ventricle, coronary artery, and cerebral blood vessels more directly and accurately, so it is more significant than the brachial artery pressure. The study found that the level of CAP was related to subclinical organ damage in the heart and is independent of the cardiovascular risk factors [44]. It showed that central but not brachial blood pressure can predict cardiovascular events in an unselected geriatric population [45]. However, guidelines considered that, despite the growing interest in some indirectly various methods, more investigation is needed before recommending the routine measurement of central blood pressure for clinical use [46].

Augmentation index (AI) was obtained from the synthesized aortic pressure waveform [41]. AI, measured using radial artery pulse wave analysis, was defined as the difference between the second and the first systolic peaks of the central arterial waveform, expressed as the percentage of central pulse pressure [47]. It provides a composite measure of aortic elasticity plus muscle artery stiffness and wave reflection [48]. Although heart rate is known to greatly influence AI, it often can be corrected for an index normalized for heart rate of 75 beats per minute [49]. A meta-analysis showed AI to be an independent predictor of cardiovascular events and all-cause mortality [50]. AI can be influenced by mean arterial pressure, age, sex, and heart rate. Different from PWV which evaluates the hardness of the arteries between two recording points, AI can quantitatively reflect the elastic of the overall arterial system [51] and has become an important index to evaluate arterial compliance. Increase of AI suggested a hardening of the arteries and is used in clinical research due to its simple and convenient measurement. It was found that AI was more predictive for cardiovascular events than systolic blood pressure, diastolic blood pressure, or pulse pressure [52].

Pulse pressure (PP), with simple and convenient measurement, is widely used as roughly reflection of arterial stiffness, and increased PP showed increased arterial stiffness [8].

The large artery elasticity index (C1) and small artery elasticity index (C2) obtained by the pulse waveform analysis of the radial artery are two of the parameters of arterial elasticity, getting through the analysis of the radial artery diastolic blood pressure pulse waveform. Studies suggested that C1 and C2 were better than the traditional blood pressure detection for clinical application of early recognition of the cardiovascular disease [53], and C1 and C2 were independently associated with cardiovascular disease [54, 55]. However, there is some doubt that radial artery derived compliance measures truly represent systemic vascular compliance [56].

Most importantly, the common limitation of CAP, AI, C1, or C2 is the lack of reference values and adequate evidence to date, therefore limiting the current widely clinical application.

## 4. Measurement of Limb Artery-Ankle Brachial Index (ABI)

ABI concept was proposed by Winsor in the 50s in twentieth Century, which was originally proposed as a noninvasive diagnostic technique for peripheral arterial disease (PAD) [57]. In 1982, ABI value less than or equal to 0.90 was identified as the diagnostic criteria of lower extremity PAD [58]. ABI is a simple, inexpensive evaluation of peripheral arterial disease and peripheral arterial stiffness and is a predictor of cardiovascular disease events [59]. The peripheral arterial disease guidelines proposed ABI normal value of 1.00–1.40 and ABI less than or equal to 0.90 as abnormal; ABI > 1.40 showed poor blood vessel elasticity [60]. Studies have shown that low ABI (ABI < 0.9) was related to the severity and degree of coronary artery disease and related to increased risk in recurrent of major cardiovascular events [61]. The risk of cardiovascular events increased independently of conventional risk factors in people with ABI equal or less than 0.90 [62]. In addition, high ABI values (>1.40 or incompressible) were correlated with greater left ventricular mass measured by cardiac magnetic resonance [63]. Furthermore, adding ABI less than or equal to 0.9 to traditional risk factors can improve the prediction value of major cardiovascular events [64]. And another meta-analysis found that ABI was able to improve the accuracy of cardiovascular risk prediction better than the Framingham risk score [65]. Thus, ABI with the advantages of low cost, simple, easy operation, and good repeatability can be used in combination with the comprehensive risk factors to screen population by noninvasive, automatic detection and analysis system of vascular apparatus. However, in people with severe arterial calcification or vascular occlusion, the value of ABI may be abnormal.

## 5. Evaluation of Carotid Atherosclerosis

Ultrasound imaging technique as a noninvasive detection for clinical evaluation of carotid plaque (CP), carotid stenosis, and carotid artery intima-media thickness carotid (CIMT) is widely used. Ultrasound detection of carotid atherosclerotic lesions can be used as the observation window of the whole vascular bed atherosclerosis and can observe early atherosclerotic lesions dynamically. Its operating convenience, low cost, and good reproducibility made it a noninvasive screening of some high-risk and asymptomatic vascular disease patients [8]. Although CIMT did not consistently improve risk classification of individuals, the study found that CIMT was a significant and independent predictor of cardiovascular events [66]. High resolution multidetector row computed tomography angiography (CTA) can accurately assess plaque composition due to its advantages of rapid examination, relatively low cost, and being capable of measuring the absolute density as well as accurate identification and quantification of calcification, but for the identification of lipid core and plaque bleeding there is still lack of specificity [67]. High resolution magnetic resonance imaging (MRI) can describe the characteristics of carotid artery plaque, identify and measure plaque composition (lipid rich core, fibrous cap thickness, plaque bleeding and calcification, etc.), and detect vulnerable plaque. Compared with other imaging techniques, MRI has very high sensitivity and specificity for the identification and detection of plaque composition. However, the high cost, more contraindications, limited availability, and checking for a long time of MRI made it unable to realize the purpose of screening [67].

Whether CIMT and CP are distinct phenotypes or represent a different stage of atherosclerotic development is unclear. Some studies indicated that common CIMT can independently predict CP occurrence and suggested that increased CIMT might occur in an earlier phase of the atherosclerotic process [68, 69]. However, another study showed that increased CIMT is not an independent predictor of CP development although these atherosclerotic phenotypes often coexist and share some common vascular determinants. The result may suggest a distinct mechanism leading to formation of incident CP, rather than a continuum of the development of CP from CIMT [70]. In addition, accumulating evidences suggest that, although CIMT and CP, two phenotypes of carotid atherosclerosis, may share some common mechanisms, their predictive power of cerebrovascular disease (CVD) risk differs. In the ARIC study, the risk of coronary heart disease can be improved by adding plaque and CIMT to the traditional risk factors [71]. Both increased CIMT and CP were useful prognosticators to predict long term future cardiovascular events [72] and were associated with increased risk of ischemic stroke [73] and can be useful predictors of prevalence and severity of coronary artery disease [74]. However, CIMT measurement alone without CP presence or CP burden measures has limited value in risk prediction [75].

An article summarizing studies on CIMT and CP showed that CP was a better marker of CVD risk than CIMT and combined CIMT with CP assessment appear better than either measure alone [76]. Meta-analysis showed that CP, compared with CIMT, had a higher diagnostic accuracy for the prediction of future coronary artery disease events and cardiovascular events [77]. Evidence supporting a role for CIMT measurement in individual patients is poor. However, assessment of CP for the presence and volume seems much more promising for directing primary prevention strategies to those who will benefit from it [78].

## 6. Coronary Artery Calcium Score (CACS)

CACS can be used for early diagnosis of coronary heart disease, review, and follow-up after treatment, screening for cardiovascular disease and high-risk population, and coronary artery CT angiography is the main noninvasive method for quantitative coronary artery calcified plaque at present [79]. CACS is commonly used to evaluate the severity of coronary artery calcification, and there are three methods: namely, Agatston integral, volume integral, and mass integral. Coronary artery calcification was quantified as a numerical value and Agatston integral was mostly used as a quantitative parameter of CACS [80]. CACS was used to evaluate subclinical disease and can improve the predictive value of cardiovascular events. Studies found that CACS was an independent predictor of coronary heart disease or cardiovascular events in the middle risk group [81]; in addition, in patients with no coronary artery calcification the future risk of cardiovascular events was very low [82].

## 7. Ambulatory Blood Pressure Monitoring (ABPM) and Dynamic Electrocardiography (ECG, Holter) Monitoring

The ambulatory blood pressure recording instrument is composed of a transducer, a miniature recording box, and a recovery system and can automatically store data within 24 hours of the day and night, including systolic blood pressure, diastolic blood pressure, mean arterial pressure, heart rate, and the highest and lowest values. The 2013 European updated guidelines on hypertension stressed the importance of ABPM for the prognosis of clinical cardiovascular events, especially for clinic blood pressure and family pressure in apparent inconsistency, night hypertension, and blood pressure variability assessment [46]. 24-hour ABPM parameters were related to arterial function parameters and can predict future cardiovascular events, especially stroke [83].

The study of dynamic electrocardiogram was initiated in 1957 by Holter and first applied in the monitoring of cardiac electrical activity, thus also called Holter ECG. It can continuously record the daily activity under the condition of 24 hours of ECG changes, is noninvasive and easy to operate, and is widely used in clinical practice. The results of Holter ECG include heart rate, arrhythmia analysis, ST-T segment analysis, and analysis of heart rate variability. Statement of clinical application of ECG and dynamic ECG in the United States made dynamic ECG monitoring an important indication [84].

## 8. Detection of Biomarkers

About early assessment of vascular disease, in addition to direct or indirect imaging techniques or noninvasive evaluation techniques for arterial stiffness, there are also many risk biomarkers of vascular disease, including traditional biomarkers and some new markers found in recent years. Basic research showed that vascular endothelial cells are an important paracrine organ and play a variety of physiological roles through the secretion or release of a series of biologically active medium, and vascular endothelial dysfunction can lead to some abnormal indicators. Therefore, the detection of biomarkers can be used as the evaluation index of endothelial dysfunction [8]. In addition, biomarkers of vascular disease, such as N-terminal probrain natriuretic peptide (NT-proBNP), high sensitivity C-reactive protein (hs-CRP), homocysteine, fibrinogen, triglycerides, uric acid, intercellular adhesion molecules-1, interleukin-6, and serum albumin, were also involved in the pathological progression of atherosclerosis [8, 85]. The study found that CAVI and NT-proBNP were higher in patients with hypertension and coronary heart disease, and the two were significantly associated [86]. Our research team previous studies also found that different disease states of population, its vascular indicators, and biological indicators were different and were influenced by many kinds of related factors. For example, hypertension, diabetes, coronary heart disease, and lower extremity arterial disease patients CAVI levels were higher and related to blood lipids, hs-CRP, NT-proBNP, and homocysteine [30–34]. For people with no known cardiovascular disease, it is found that the use of CRP or fibrinogen to assess risk can be helpful for the prevention of cardiovascular events [87]. A consensus panel presented that these new clinical examinations such as glycosylated hemoglobin, trace urinary albumin, C-reactive protein, lipoprotein associated phospholipase, coronary artery calcification, carotid intima-media thickness, and ankle brachial index can be used in cardiovascular risk prediction [88]. The American Heart Association also recommends that, in addition to traditional risk factors, family history, glycosylated hemoglobin, and microalbumin urine can be used to predict the risk of coronary heart disease [25]. A study including 30 markers finally screened 3 markers, namely, N-terminal probrain natriuretic peptide, C-reactive protein, and sensitive troponin I. Adding these three markers to the traditional risk prediction model can improve the predictive value of cardiovascular events in the future 10 years [89]. A follow-up study found that coronary heart disease can reduce the incidence rate of 66% mainly attributed to the change of risk factors, the favorable changes in cholesterol accounted for 32%, and blood pressure, smoking, and exercise were 14%, 13%, and 9%, respectively [90]. Therefore, the identification of new vascular related biomarkers and early intervention can reduce the incidence of vascular disease.

## 9. Genetic Evaluation

Arteriosclerosis is not the patent of the modern society, its existence precedes the modern civilized society and the present risk factor [91], and traditional risk factors just are only part of arterial stiffness and genetic factors also occupy a part. The study, which was found by CT scans of the mummy in 3300 BC, was the first document of the carotid artery atherosclerosis, which represented the earliest records of human atherosclerosis [92]. Study found that atherosclerosis was actually the basis of aging, early after began to degenerate early after birth and progress with age. The interaction between genes and environment played an important role in the occurrence and development of atherosclerosis, genes created the vulnerability of atherosclerosis, and whether or when clinical atherosclerosis occurs was determined by environment [93].

Hyperhomocysteinemia (HHcy) is characterized by elevated plasma total homocysteine levels and associated with increased risk of cardiovascular disease and stroke [94]. Studies have found that Hcy and CAVI were significantly positively correlated in patients with vascular disease [31], and HHcy patients endothelial function was impaired [95]. Methylenetetrahydrofolate reductase (MTHFR) is a key enzyme in the folate pathway and can mediate the clearance of homocysteine in human body. The genetic polymorphisms of CT and TT in the 677th genes of MTHFR gene resulted in the change of MTHFR enzyme activity, leading to metabolic disturbance of folic acid. Studies showed that C677T gene polymorphism can be used as a useful screening marker for severe HHcy [96]. The relationship between metalloproteinase matrix (MMP) family gene polymorphism and coronary heart disease (CHD) has been widely studied, and genetic defects lead to MMPs activation overexpression playing a key role in the pathogenesis of CHD. Studies indicated that extracellular matrix metabolism regulation abnormalities play a substantial role in vascular remodeling [97]. MicroRNAs (miRNAs) are an important regulator of endothelial function by fine tuning of gene expression. The specific expression of miRNAs combined together can be used to identify the developmental stages of disease, and intrinsic biological information obtained by miRNAs may be transformed into a new treatment method [98]. It was found that the gene variant of apolipoprotein E gene is the strongest signal of low density lipoprotein cholesterol and also related to cardiovascular risk factors such as high density lipoprotein cholesterol, triglycerides, inflammatory biomarkers C-reactive protein, and CIMT [99]. The peroxisome proliferator-activated receptor (PPARG) is a nuclear hormone receptor and plays an important role in obesity, insulin resistance, and other metabolic diseases as well as CHD. PPARG has two common genetic polymorphisms, namely, P12A and C161T. Recent studies showed that both of these two polymorphisms may affect individual susceptibility to metabolic disease and CHD risk, but there have also been studies which found that C161T PPARG slightly increased the sensitivity of coronary heart disease, and the P12A PPARG was not associated with the risk of CHD [100]. In a study, genetic risk score based on 13 single nucleotide polymorphisms associated with coronary heart disease can be able to identify 20% of the individuals in the European pedigree with an increased risk of coronary heart disease events by 70% [101]. Therefore, it will be possible to provide theoretical basis for gene targeted therapy by using genetic evaluation method to detect the genetic loci of vascular lesions.

All the technologies of vascular early detection mentioned above were summarized within a table (see Table 1), including the region of interest, advantages, and disadvantages about all kinds of measurement for vascular health.

## 10. Establishment and Prospect of Comprehensive Evaluation System of Vascular Disease

Based on the preliminary research foundation, in 2006, our research team released the first report of Chinese vascular disease early detection technology application guide [102]. The guideline covered a variety of effective indicators for early assessment of vascular disease. Then according to the 2006 guideline recommendation, we conducted a series of studies, including the vascular function in different diseases, different vascular assessment techniques and biomarker research, and drug intervention studies. After further researches, the guideline was updated in 2011, and some new vascular assessment techniques were added [26]. The evaluation system combined with a variety of assessment techniques from different levels of vascular structure and function, such as biomarkers, imaging indexes, and noninvasive arterial stiffness. There were relevant recommendations published by the European updated guidelines in 2013; for example, CIMT was recommended, CF-PWV was used to evaluate arterial stiffness, and ABI can be used to assess peripheral arterial disease [46]. Similarly, the United States also published a guideline and recommended that the evaluation of coronary artery calcium score, ABI, family history, or high sensitivity C reactive protein was considerable when it was based on treatment decisions; however, it is not sure whether the carotid IMT can be used as a routine clinical examination to assess the risk of a first cardiovascular event [103].

At present, there are a variety of cardiovascular risk prediction models [104], to predict the risk of cardiovascular events in the future, with the famous Framingham scoring system and Reynolds risk assessment system. However, these scoring systems are assessing the traditional risk factors for cardiovascular risk, such as age, sex, body mass index, smoking, family history, systolic blood pressure, diabetes, total cholesterol, high density lipoprotein cholesterol, and high sensitivity C-reactive protein, and did not directly assess the blood vessels that cause disease. In addition, these models predict the risk of cardiovascular disease over the next 10 years or even longer, while there is lack of a recent risk assessment in order to carry out interventions and prevent the occurrence of events. Furthermore, present assessment of vascular health status, limited to a single indicator, although there are multiple indicators, did not carry out effective combination and even classification of various indicators. Therefore, the new vascular health classification, on the basis of traditional risk factors, superimposed the vascular structure and function evaluation indicators recommended in the guidelines, directly regarding the blood vessel as the target, and conducted the grading management, to comprehensively assess the vascular health status, which also reflects the current hot topic, precision medicine [38].

## Figures and Tables

**Table 1:** Summarizing early evaluation system of vascular health.

Techniques	Region of interest	Advantages	Disadvantages
*Vascular endothelial function*	Vascular endothelium		
Coronary angiography [7]	Coronary artery	Gold standard	Invasive, difficult, time consuming with high cost
FMD [17, 18, 20, 21]	Conduit brachial artery	Noninvasive, simple, most used	Technical requirements, Multiple influencing factors
PAT-RHI [18, 20, 21]	Finger microvasculature	Automatic detection, simple operation	More data on predictive value
*Arterial stiffness*			
PWV [22, 23]	Large artery function	Gold standard	Dependent on blood pressure
CAVI [29, 30]	Large artery function and structure	Independent of blood pressure	Influenced by lower ABI value, more data on predictive value
CAP [39–43]	Cardiac catheterization	Directly	Invasive
	Systolic radial or carotid artery waveforms	Noninvasive, easy conduction	Need more investigation
AI [41, 47, 50, 51]	Systolic radial or carotid artery waveforms analysis	Noninvasive, automatically detected	Lack of reference value, need more investigation
C1/C2 [53–55]	Diastolic radial artery waveform analysis	Noninvasive, automatically detected	Lack of reference value, need more investigation
*ABI* [59, 60, 65]	Peripheral limb artery	Gold standard of PAD, predictor of CVD, easy operation, good repeatability	Not suitable for people with severe arterial calcification or vascular occlusion
*Carotid atherosclerosis*			
CIMT [75, 76, 78]	Structure of carotid artery	Noninvasive, easy operation	Multiple location of measurements
CP [75, 76]	Structure of carotid artery	Noninvasive, easy operation	Plaque multiple variation
*CACS* [79, 80]	Coronary artery structure	High cost	Prediction of CVD
*ABPM and Holter* [46, 83, 84]	Ambulatory monitoring of blood pressure and ECG	Low cost, real-time monitoring	Prognosis of CVD
*Biomarkers* [25, 88, 89]	Blood indicators	Simple, inexpensive, good reproducibility	Need further validation
*Genetic evaluation* [91–93]	Genetic loci	Gene targeted therapy	Need further validation

## References

[1] Lloyd-Jones D. M., Hong Y., Labarthe D. (2010). Defining and setting national goals for cardiovascular health promotion and disease reduction: the American Heart Association's strategic impact goal through 2020 and beyond. *Circulation*.

[2] Chen W., Gao R., Liu L. (2015). Chinese cardiovascular disease report 2014. *Chinese Circulation Journal*.

[3] Wang H.-Y. (2014). Inauguration and trend of development of discipline of vascular medicine in China. *Medical Journal of Chinese People's Liberation Army*.

[4] Cahill P. A., Redmond E. M. (2016). Vascular endothelium—gatekeeper of vessel health. *Atherosclerosis*.

[5] Vita J. A. (2011). Endothelial function. *Circulation*.

[6] Della Rocca D. G., Pepine C. J. (2010). Endothelium as a predictor of adverse outcomes. *Clinical Cardiology*.

[7] Ludmer P. L., Selwyn A. P., Shook T. L. (1986). Paradoxical vasoconstriction induced by acetylcholine in atherosclerotic coronary arteries. *The New England Journal of Medicine*.

[8] Wang H. (2011). Chinese guideline for early vascular disease detection (Second report of 2011). *Advances in Cardiovascular Diseases*.

[9] Yeboah J., Folsom A. R., Burke G. L. (2009). Predictive value of brachial flow-mediated dilation for incident cardiovascular events in a population-based study: the multi-ethnic study of atherosclerosis. *Circulation*.

[10] Yang Y., Xu J.-Z., Wang Y., Tang X.-F., Gao P.-J. (2014). Brachial flow-mediated dilation predicts subclinical target organ damage progression in essential hypertensive patients: a 3-year follow-up study. *Journal of Hypertension*.

[11] Rubinshtein R., Kuvin J. T., Soffler M. (2010). Assessment of endothelial function by non-invasive peripheral arterial tonometry predicts late cardiovascular adverse events. *European Heart Journal*.

[12] Akiyama E., Sugiyama S., Matsuzawa Y. (2012). Incremental prognostic significance of peripheral endothelial dysfunction in patients with heart failure with normal left ventricular ejection fraction. *Journal of the American College of Cardiology*.

[13] Matsuzawa Y., Sugiyama S., Sumida H. (2013). Peripheral endothelial function and cardiovascular events in high-risk patients. *Journal of the American Heart Association*.

[14] Matsuzawa Y., Sugiyama S., Sugamura K. (2010). Digital assessment of endothelial function and ischemic heart disease in women. *Journal of the American College of Cardiology*.

[15] Matsuzawa Y., Li J., Aoki T. (2015). Predictive value of endothelial function by non-invasive peripheral arterial tonometry for coronary artery disease. *Coronary Artery Disease*.

[16] Anderson T. J., Phillips S. A. (2015). Assessment and prognosis of peripheral artery measures of vascular function. *Progress in Cardiovascular Diseases*.

[17] Xu Y., Arora R. C., Hiebert B. M. (2014). Non-invasive endothelial function testing and the risk of adverse outcomes: a systematic review and meta-analysis. *European Heart Journal Cardiovascular Imaging*.

[18] Matsuzawa Y., Kwon T. G., Lennon R. J., Lerman L. O., Lerman A. (2015). Prognostic value of flow-mediated vasodilation in brachial artery and fingertip artery for cardiovascular events: a systematic review and meta-analysis. *Journal of the American Heart Association*.

[19] Nohria A., Gerhard-Herman M., Creager M. A., Hurley S., Mitra D., Ganz P. (2006). Role of nitric oxide in the regulation of digital pulse volume amplitude in humans. *Journal of Applied Physiology*.

[20] Hamburg N. M., Palmisano J., Larson M. G. (2011). Relation of brachial and digital measures of vascular function in the community: the framingham heart study. *Hypertension*.

[21] Schnabel R. B., Schulz A., Wild P. S. (2011). Noninvasive vascular function measurement in the community: cross-sectional relations and comparison of methods. *Circulation: Cardiovascular Imaging*.

[22] Lakatta E. G. (2003). Arterial and cardiac aging: major shareholders in cardiovascular disease enterprises. Part III: cellular and molecular clues to heart and arterial aging. *Circulation*.

[23] Boutouyrie P., Fliser D., Goldsmith D. (2014). Assessment of arterial stiffness for clinical and epidemiological studies: methodological considerations for validation and entry into the European Renal and Cardiovascular Medicine registry. *Nephrology Dialysis Transplantation*.

[24] Coutinho T., Turner S. T., Kullo I. J. (2011). Aortic pulse wave velocity is associated with measures of subclinical target organ damage. *JACC: Cardiovascular Imaging*.

[25] Mitchell G. F., Hwang S.-J., Vasan R. S. (2010). Arterial stiffness and cardiovascular events: the Framingham Heart Study. *Circulation*.

[26] Mattace-Raso F. U. S., van der Cammen T. J. M., Hofman A. (2006). Arterial stiffness and risk of coronary heart disease and stroke: the Rotterdam Study. *Circulation*.

[27] Wilkinson I. B., Cockcroft J. R., McEniery C. M. (2015). Aortic stiffness as a cardiovascular risk predictor. *British Medical Journal*.

[28] Gasecki D., Rojek A., Kwarciany M. (2012). Aortic stiffness predicts functional outcome in patients after ischemic stroke. *Stroke*.

[29] Hayashi K., Yamamoto T., Takahara A., Shirai K. (2015). Clinical assessment of arterial stiffness with cardio-ankle vascular index: theory and applications. *Journal of Hypertension*.

[30] Wang H. (2015). Cardio-ankle vascular index: a new marker for vascular health evaluation (experience from China). *Journal of Human Hypertension*.

[31] Wang H., Liu J., Wang Q. (2013). Descriptive study of possible link between cardioankle vascular index and homocysteine in vascular-related diseases. *BMJ Open*.

[32] Wang H., Liu J., Zhao H. (2013). Arterial stiffness evaluation by cardio-ankle vascular index in hypertension and diabetes mellitus subjects. *Journal of the American Society of Hypertension*.

[33] Wang H., Shirai K., Liu J. (2014). Comparative study of cardio-ankle vascular index between Chinese and Japanese healthy subjects. *Clinical and Experimental Hypertension*.

[34] Wang H., Liu J., Zhao H. (2015). Relationship between cardio-ankle vascular index and plasma lipids in hypertension subjects. *Journal of Human Hypertension*.

[35] Nam S. H., Kang S. G., Lee Y. A. (2015). Association of metabolic syndrome with the cardio ankle vascular index in asymptomatic Korean population. *Journal of Diabetes Research*.

[36] Sugimoto T., Misu S., Sawa R. (2016). Association between the cardio-ankle vascular index and executive function in community-dwelling elderly people. *Journal of Atherosclerosis and Thrombosis*.

[37] Satoh-Asahara N., Kotani K., Yamakage H. (2015). Cardio-ankle vascular index predicts for the incidence of cardiovascular events in obese patients: a multicenter prospective cohort study (Japan Obesity and Metabolic Syndrome Study: JOMS). *Atherosclerosis*.

[38] Wang H., Liu H. (2015). A new classification of vascular health and vascular medicine. *Advances in Cardiovascular Diseases*.

[39] O'Rourke M. F., Adji A. (2012). Noninvasive studies of central aortic pressure. *Current Hypertension Reports*.

[40] Cloud G. C., Rajkumar C., Kooner J., Cooke J., Bulpitt C. J. (2003). Estimation of central aortic pressure by SphygmoCor® requires intra-arterial peripheral pressures. *Clinical Science*.

[41] Chen C.-H., Nevo E., Fetics B. (1997). Estimation of central aortic pressure waveform by mathematical transformation of radial tonometry pressure: validation of generalized transfer function. *Circulation*.

[42] O'Rourke M. F., Seward J. B. (2006). Central arterial pressure and arterial pressure pulse: new views entering the second century after Korotkov. *Mayo Clinic Proceedings*.

[43] Takazawa K., Kobayashi H., Shindo N., Tanaka N., Yamashina A. (2007). Relationship between radial and central arterial pulse wave and evaluation of central aortic pressure using the radial arterial pulse wave. *Hypertension Research*.

[44] Cui R., Li Y., Krisztina G. (2014). An association between central aortic pressure and subclinical organ damage of the heart among a general Japanese cohort: Circulatory Risk in Communities Study (CIRCS). *Atherosclerosis*.

[45] Pini R., Cavallini M. C., Palmieri V. (2008). Central but not brachial blood pressure predicts cardiovascular events in an unselected geriatric population. The ICARe dicomano study. *Journal of the American College of Cardiology*.

[46] ESH/ESC Task Force for the Management of Arterial Hypertension (2013). 2013 Practice guidelines for the management of arterial hypertension of the European Society of Hypertension (ESH) and the European Society of Cardiology (ESC): ESH/ESC Task Force for the Management of Arterial Hypertension. *Journal of Hypertension*.

[47] Laurent S., Cockcroft J., Van Bortel L. (2006). Expert consensus document on arterial stiffness: methodological issues and clinical applications. *European Heart Journal*.

[48] Safar M. E., London G. M. (2000). Therapeutic studies and arterial stiffness in hypertension: recommendations of the European Society of Hypertension. *Journal of Hypertension*.

[49] Wilkinson I. B., MacCallum H., Flint L., Cockcroft J. R., Newby D. E., Webb D. J. (2000). The influence of heart rate on augmentation index and central arterial pressure in humans. *Journal of Physiology*.

[50] Vlachopoulos C., Aznaouridis K., O'Rourke M. F., Safar M. E., Baou K., Stefanadis C. (2010). Prediction of cardiovascular events and all-cause mortality with central haemodynamics: a systematic review and meta-analysis. *European Heart Journal*.

[51] Riggio S., Mandraffino G., Sardo M. A. (2010). Pulse wave velocity and augmentation index, but not intima-media thickness, are early indicators of vascular damage in hypercholesterolemic children. *European Journal of Clinical Investigation*.

[52] Kim D. H., Braam B. (2013). Assessment of arterial stiffness using applanation tonometry. *Canadian Journal of Physiology and Pharmacology*.

[53] Brumback L. C., Jacobs D. R., Dermond N., Chen H., Duprez D. A. (2010). Reproducibility of arterial elasticity parameters derived from radial artery diastolic pulse contour analysis: the multi-ethnic study of atherosclerosis. *Blood Pressure Monitoring*.

[54] Duprez D. A., Jacobs D. R., Lutsey P. L. (2009). Large and small artery elasticity, but not coronary calcium score and carotid intima-media thickness, predict congestive heart failure events in an asymptomatic population: results of the Multiethnic Study of Atherosclerosis (MESA). *JACC*.

[55] Duprez D. A., Jacobs D. R., Lutsey P. L. (2009). Small artery but not large artery elasticity predicts coronary heart disease events beyond coronary calcium score and carotid intima-media thickness in an asymptomatic population: results of the Multi-Ethnic Study of Atherosclerosis (MESA). *Journal of the American College of Cardiology*.

[56] Manning T. S., Shykoff B. E., Izzo J. L. (2002). Validity and reliability of diastolic pulse contour analysis (Windkessel model) in humans. *Hypertension*.

[57] Winsor T. (1950). Influence of arterial disease on the systolic blood pressure gradients of the extremity. *The American Journal of the Medical Sciences*.

[58] Ouriel K., McDonnell A. E., Metz C. E., Zarins C. K. (1982). Critical evaluation of stress testing in the diagnosis of peripheral vascular disease. *Surgery*.

[59] Moyer V. A. (2013). Screening for peripheral artery disease and cardiovascular disease risk assessment with the ankle-brachial index in adults: U.S. preventive services task force recommendation statement. *Annals of Internal Medicine*.

[60] Anderson J. L., Halperin J. L., Albert N. M. (2013). Management of Patients With Peripheral Artery Disease (Compilation of 2005 and 2011 ACCF/AHA Guideline Recommendations): a report of the American College of Cardiology Foundation/American Heart Association Task Force on Practice Guidelines. *Journal of the American College of Cardiology*.

[61] Papa E. D. E., Helber I., Ehrlichmann M. R. (2013). Ankle-brachial index as a predictor of coronary disease events in elderly patients submitted to coronary angiography. *Clinics*.

[62] Kojima I., Ninomiya T., Hata J. (2014). A low ankle brachial index is associated with an increased risk of cardiovascular disease: the hisayama study. *Journal of Atherosclerosis and Thrombosis*.

[63] Ix J. H., Katz R., Peralta C. A. (2010). A high ankle brachial index is associated with greater left ventricular mass. MESA (Multi-Ethnic Study of Atherosclerosis). *Journal of the American College of Cardiology*.

[64] Velescu A., Clara A., Peñafiel J. (2015). Adding low ankle brachial index to classical risk factors improves the prediction of major cardiovascular events. The REGICOR study. *Atherosclerosis*.

[65] Fowkes F. G. R., Murray G. D., Butcher I. (2008). Ankle brachial index combined with Framingham risk score to predict cardiovascular events and mortality: a meta-analysis. *The Journal of the American Medical Association*.

[66] Lorenz M. W., Schaefer C., Steinmetz H., Sitzer M. (2010). Is carotid intima media thickness useful for individual prediction of cardiovascular risk? Ten-year results from the Carotid Atherosclerosis Progression Study (CAPS). *European Heart Journal*.

[67] Naim C., Douziech M., Therasse É. (2014). Vulnerable atherosclerotic carotid plaque evaluation by ultrasound, computed tomography angiography, and magnetic resonance imaging: an overview. *Canadian Association of Radiologists Journal*.

[68] Zureik M., Ducimetière P., Touboul P.-J. (2000). Common carotid intima-media thickness predicts occurrence of carotid atherosclerotic plaques: longitudinal results from the aging vascular study (EVA) study. *Arteriosclerosis, Thrombosis, and Vascular Biology*.

[69] von Sarnowski B., Lüdemann J., Völzke H., Dörr M., Kessler C., Schminke U. (2010). Common carotid intima-media thickness and framingham risk score predict incident carotid atherosclerotic plaque formation: longitudinal results from the study of health in Pomerania. *Stroke*.

[70] Rundek T., Gardener H., Della-Morte D. (2015). The relationship between carotid intima-media thickness and carotid plaque in the Northern Manhattan Study. *Atherosclerosis*.

[71] Nambi V., Chambless L., Folsom A. R. (2010). Carotid intima-media thickness and presence or absence of plaque improves prediction of coronary heart disease risk: the ARIC (Atherosclerosis Risk In Communities) study. *Journal of the American College of Cardiology*.

[72] Lee S., Cho G.-Y., Kim H.-S. (2014). Common carotid intima-media thickness as a risk factor for outcomes in Asian patients with acute ST-elevation myocardial infarction. *Canadian Journal of Cardiology*.

[73] Bekwelem W., Jensen P. N., Norby F. L. (2016). Carotid Atherosclerosis and stroke in atrial fibrillation: the Atherosclerosis Risk in Communities study. *Stroke*.

[74] Jeevarethinam A., Venuraju S., Weymouth M., Atwal S., Lahiri A. (2015). Carotid intimal thickness and plaque predict prevalence and severity of coronary atherosclerosis: a pilot study. *Angiology*.

[75] Ho S. S. (2016). Current status of carotid ultrasound in atherosclerosis. *Quantitative Imaging in Medicine and Surgery*.

[76] Naqvi T. Z., Lee M.-S. (2014). Carotid intima-media thickness and plaque in cardiovascular risk assessment. *JACC: Cardiovascular Imaging*.

[77] Inaba Y., Chen J. A., Bergmann S. R. (2012). Carotid plaque, compared with carotid intima-media thickness, more accurately predicts coronary artery disease events: a meta-analysis. *Atherosclerosis*.

[78] Sillesen H. (2014). Carotid intima-media thickness and/or carotid plaque: what is relevant?. *European Journal of Vascular and Endovascular Surgery*.

[79] Vliegenthart R., Morris P. B. (2012). Computed tomography coronary artery calcium scoring: review of evidence base and cost-effectiveness in cardiovascular risk prediction. *Journal of Thoracic Imaging*.

[80] Agatston A. S., Janowitz W. R., Hildner F. J., Zusmer N. R., Viamonte M., Detrano R. (1990). Quantification of coronary artery calcium using ultrafast computed tomography. *Journal of the American College of Cardiology*.

[81] Yeboah J., McClelland R. L., Polonsky T. S. (2012). Comparison of novel risk markers for improvement in cardiovascular risk assessment in intermediate-risk individuals. *The Journal of the American Medical Association*.

[82] Sarwar A., Shaw L. J., Shapiro M. D. (2009). Diagnostic and prognostic value of absence of coronary artery calcification. *JACC: Cardiovascular Imaging*.

[83] Kollias A., Stergiou G. S., Dolan E., O'Brien E. (2012). Ambulatory arterial stiffness index: a systematic review and meta-analysis. *Atherosclerosis*.

[84] Kadish A. H., Buxton A. E., Kennedy H. L. (2001). ACC/AHA clinical competence statement on electrocardiography and ambulatory electrocardiography: a report of the ACC/AHA/ACP-ASIM task force on clinical competence (ACC/AHA Committee to Develop a Clinical Competence Statement on Electrocardiography and Ambulatory Electrocardiography). *Journal of the American College of Cardiology*.

[85] Giles T. (2013). Biomarkers, cardiovascular disease, and hypertension. *Journal of Clinical Hypertension*.

[86] Wang H., Liu J., Zhao H. (2014). Relationship between cardio-ankle vascular index and N-terminal pro-brain natriuretic peptide in hypertension and coronary heart disease subjects. *Journal of the American Society of Hypertension*.

[87] Emerging Risk Factors Collaboration, Kaptoge S., Di Angelantonio E. (2012). C-reactive protein, fibrinogen, and cardiovascular disease prediction. *The New England Journal of Medicine*.

[88] Melander O., Newton-Cheh C., Almgren P. (2009). Novel and conventional biomarkers for prediction of incident cardiovascular events in the community. *Journal of the American Medical Association*.

[89] Blankenberg S., Zeller T., Saarela O. (2010). Contribution of 30 biomarkers to 10-year cardiovascular risk estimation in 2 population cohorts: the MONICA, risk, genetics, archiving, and monograph (MORGAM) biomarker project. *Circulation*.

[90] Mannsverk J., Wilsgaard T., Mathiesen E. B. (2016). Trends in modifiable risk factors are associated with declining incidence of hospitalized and nonhospitalized acute coronary heart disease in a population. *Circulation*.

[91] Thompson R. C., Allam A. H., Lombardi G. P. (2013). Atherosclerosis across 4000 years of human history: the Horus study of four ancient populations. *The Lancet*.

[92] Murphy W. A., Zur Nedden D., Gostner P., Knapp R., Recheis W., Seidler H. (2003). The iceman: discovery and imaging. *Radiology*.

[93] Clarke E. M., Thompson R. C., Allam A. H. (2014). Is atherosclerosis fundamental to human aging? Lessons from ancient mummies. *Journal of Cardiology*.

[94] Homocysteine Studies Collaboration (2002). Homocysteine and risk of ischemic heart disease and stroke: a meta-analysis. *The Journal of the American Medical Association*.

[95] He L., Zeng H., Li F. (2010). Homocysteine impairs coronary artery endothelial function by inhibiting tetrahydrobiopterin in patients with hyperhomocysteinemia. *American Journal of Physiology—Endocrinology and Metabolism*.

[96] Wang Y., Xu X., Huo Y. (2015). Predicting hyperhomocysteinemia by methylenetetrahydrofolate reductase C677T polymorphism in Chinese patients with hypertension. *Clinical and Applied Thrombosis/Hemostasis*.

[97] Niu W., Qi Y. (2012). Matrix metalloproteinase family gene polymorphisms and risk for coronary artery disease: systematic review and meta-analysis. *Heart*.

[98] Menghini R., Casagrande V., Federici M. (2013). MicroRNAs in endothelial senescence and atherosclerosis. *Journal of Cardiovascular Translational Research*.

[99] Khan T. A., Shah T., Prieto D. (2013). Apolipoprotein E genotype, cardiovascular biomarkers and risk of stroke: systematic review and meta-analysis of 14,015 stroke cases and pooled analysis of primary biomarker data from up to 60,883 individuals. *International Journal of Epidemiology*.

[100] Ding S., Liu L., Zhuge Q.-C. (2012). The meta-analysis of the association of PPARG P12A, C161T polymorphism and coronary heart disease. *Wiener Klinische Wochenschrift*.

[101] Ripatti S., Tikkanen E., Orho-Melander M. (2010). A multilocus genetic risk score for coronary heart disease: Case-control and prospective cohort analyses. *The Lancet*.

[102] Wang H. (2006). Chinese guideline for early vascular disease detection (first report). *Medical Journal of Chinese People's Health*.

[103] Goff D. C., Lloyd-Jones D. M., Bennett G. (2014). 2013 ACC/AHA guideline on the assessment of cardiovascular risk: a report of the American college of cardiology/American heart association task force on practice guidelines. *Circulation*.

[104] Allan G. M., Nouri F., Korownyk C., Kolber M. R., Vandermeer B., McCormack J. (2013). Agreement among cardiovascular disease risk calculators. *Circulation*.

